# AI-Driven Polypharmacology in Small-Molecule Drug Discovery

**DOI:** 10.3390/ijms26146996

**Published:** 2025-07-21

**Authors:** Mena Abdelsayed

**Affiliations:** Lankenau Institute for Medical Research, 100 E Lancaster Ave., Penn Wynne, PA 19096, USA; abdelsayedm@mlhs.org

**Keywords:** polypharmacology, multi-target drug design, artificial intelligence (AI), deep learning, network pharmacology, generative models, computational drug discovery

## Abstract

Polypharmacology, the rational design of small molecules that act on multiple therapeutic targets, offers a transformative approach to overcome biological redundancy, network compensation, and drug resistance. This review outlines the scientific rationale for polypharmacology, highlighting its success across oncology, neurodegeneration, metabolic disorders, and infectious diseases. Emphasis is placed on how polypharmacological agents can synergize therapeutic effects, reduce adverse events, and improve patient compliance compared to combination therapies. We also explore how computational methods—spanning ligand-based modeling, structure-based docking, network pharmacology, and systems biology—enable target selection and multi-target ligand prediction. Recent advances in artificial intelligence (AI), particularly deep learning, reinforcement learning, and generative models, have further accelerated the discovery and optimization of multi-target agents. These AI-driven platforms are capable of de novo design of dual and multi-target compounds, some of which have demonstrated biological efficacy in vitro. Finally, we discuss the integration of omics data, CRISPR functional screens, and pathway simulations in guiding multi-target design, as well as the challenges and limitations of current AI approaches. Looking ahead, AI-enabled polypharmacology is poised to become a cornerstone of next-generation drug discovery, with potential to deliver more effective therapies tailored to the complexity of human disease.

## 1. Introduction

### 1.1. The One-Target–One-Drug Paradigm and Its Limitations

**Figure 1:**
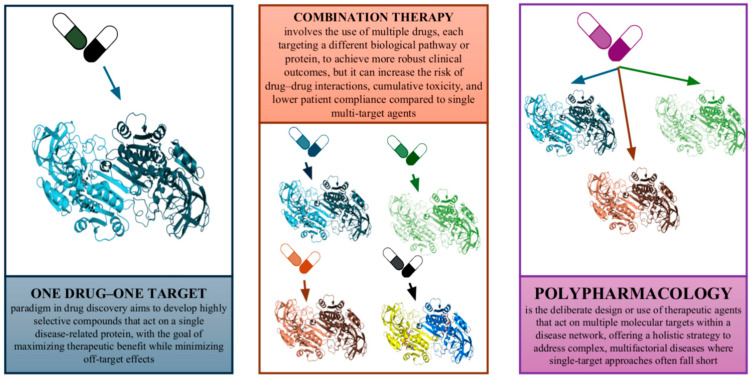
The evolution of drug discovery paradigms. A simplified diagram illustrating the shift from the classical “one drug–one target–one disease” approach to modern polypharmacology. The left side shows a single drug targeting a single protein; the right side shows a single drug interacting with multiple targets, and the middle includes combination therapy (multiple drugs targeting multiple proteins). The diagram visually contrasts the limitations of the reductionist approach with the systems/network-based rationale for polypharmacology.

For much of the past century, drug discovery was dominated by a “one target–one drug” approach, focused on developing highly selective ligands (“magic bullets”) for individual disease proteins, based on the belief that this would maximize therapeutic benefit and minimize off-target effects [1,2]. While this strategy achieved some successes, it has major limitations: many complex diseases remain unresponsive to single-target drugs, with about 90% of such candidates failing in late-stage trials due to a lack of efficacy or unexpected toxicity [3,4]. These failures often stem from the reductionist oversight of the complex, redundant, and networked nature of human biology [4,5]. In diseases with multifactorial etiologies, targeting a lone node in a complex network can easily be circumvented by the system, leading to a lack of long-term efficacy or the emergence of resistance. Indeed, the classical “one gene, one drug, one disease” hypothesis has been increasingly challenged by modern biomedical research, which shows that biological systems can adapt and compensate when a single pathway is perturbed (Figure 1) [2,5].

Historically, the preference for highly selective drugs was also driven by safety concerns—the desire to minimize “off-target” interactions that could cause side effects. Paradoxically, many effective medications were later found to be “promiscuous” in their action, hitting multiple targets [1,6]. Such drugs were sometimes pejoratively termed “dirty drugs,” yet their clinical success suggested that a certain degree of multi-target activity could be advantageous [1,7]. Furthermore, the practice of combination therapy (polypharmacy) in fields like oncology and infectious disease has long implicitly acknowledged that modulating multiple biological targets or pathways is often necessary for robust clinical outcomes. However, combination regimens with multiple drugs come with their own drawbacks: increased risk of drug–drug interactions, cumulative toxicity, complex dosing schedules, and lower patient compliance [8,9]. These issues underscore the need for a new paradigm in drug discovery—one that embraces polypharmacology in a deliberate and rational way.

### 1.2. Rationale for Polypharmacology and Multi-Target Drug Design

Polypharmacology can be advantageous in treating complex disorders, as simultaneously modulating several pathways may yield synergistic therapeutic effects greater than single-target approaches [3,10]. By addressing several key disease drivers simultaneously, a multi-target drug can enhance efficacy in diseases where a single-pathway intervention is insufficient. For example, many approved kinase inhibitors in cancer therapy owe their clinical effectiveness to a broad target profile—they inhibit multiple kinases in oncogenic signaling cascades, thereby blocking cancer cell growth through parallel routes [11,12]. In such cases, a multi-target profile helps prevent the tumor from simply “rerouting” signaling to escape a solitary blockade.

Another compelling argument for polypharmacology is the mitigation of drug resistance. Pathogens and cancer cells frequently develop resistance to highly specific drugs by acquiring mutations in the drug’s target [13]. A drug that inhibits several unrelated targets could substantially lower the probability that a single genetic change confers full resistance, since the organism would need to simultaneously adapt to multiple inhibitory actions [4]. This principle is analogous to combination therapy but is achieved with one molecule. Additionally, multi-target agents may allow lower dosing for each target than would be required for a strictly single-target drug, potentially reducing dose-dependent adverse effects [14]. By distributing pharmacological activity across multiple pathways, such an agent can produce the desired therapeutic outcome without excessively pushing any single target to the point of toxicity [4]. Early examples in the literature noted that moderately potent “promiscuous” compounds could be safer and more efficacious than a highly potent selective compound, because the former can be used at lower doses and avoid triggering off-target toxicities [7]. Moreover, if designed carefully, a multi-target drug can be engineered to avoid antagonizing off-targets that are unrelated to the disease (reducing side effects), while intentionally engaging multiple disease-relevant targets (improving efficacy) [4]. This selective polypharmacology—sometimes termed a “magic shotgun” approach—offers a holistic strategy to restore perturbed network homeostasis in complex maladies [1].

From a clinical perspective, a single polypharmacological agent also offers practical benefits in terms of patient compliance and pharmacokinetics. Combining the activities of two or three drugs into one molecule simplifies treatment regimens [2,9]. Patients take one pill instead of several, which improves adherence, particularly in elderly populations who often struggle with polypharmacy. A multi-target drug guarantees that all its activities are delivered in a fixed ratio, reaching the targets simultaneously in the correct balance. This can avoid the pharmacokinetic variability that arises when separate drugs with different absorption and elimination profiles are used in combination [9]. Notably, a single multi-target ligand will not interact with itself the way multiple co-administered drugs might, thereby eliminating drug–drug interaction risks inherent in combination therapies [8,9]. These advantages make a strong scientific and clinical case for embracing rational polypharmacology in modern drug discovery, especially as we confront diseases that defy simple, single-target solutions.

### 1.3. Complex Diseases and the Need for Multi-Target Therapeutics

The insufficiency of one-target therapies is most evident in complex, multifactorial diseases. In recent years, researchers have identified numerous conditions where a multi-target approach is not only advantageous but arguably necessary for meaningful clinical progress. We highlight several key disease areas that exemplify the need for polypharmacology, including—but not limited to—cancer, neurodegenerative disorders, metabolic and endocrine diseases, and infectious diseases.

#### 1.3.1. Cancer

Cancer is a complex, polygenic disease that activates multiple redundant signaling pathways, enabling tumors to evade single-target inhibitors. While combination chemotherapy is common, an alternative is to develop single agents that act on several oncogenic targets. Many modern anticancer drugs, such as sorafenib and sunitinib, are multi-kinase inhibitors that suppress tumor growth and delay resistance by blocking multiple pathways. Polypharmacology is especially advantageous in cancers driven by intricate networks (e.g., PI3K/Akt/mTOR), as multi-target agents can induce synthetic lethality and prevent compensatory mechanisms, resulting in more durable responses. Notably, many FDA-approved cancer drugs display polypharmacology, reflecting a shift in oncology drug design toward targeting cancer’s network biology [11,12].

#### 1.3.2. Neurodegenerative Disorders

Neurodegenerative diseases like Alzheimer’s (AD) and Parkinson’s (PD) involve complex pathological processes, including β-amyloid accumulation, tau hyperphosphorylation, oxidative stress, neuroinflammation, and neurotransmitter deficits. Single-target therapies have largely failed in AD, prompting a shift toward multi-target strategies such as combination therapies and multi-target-directed ligands (MTDLs), which integrate activities like cholinesterase inhibition and antioxidant or anti-amyloid effects within one molecule [2,15,16,17]. For example, the MTDL “memoquin” was designed to inhibit acetylcholinesterase and combat β-amyloid aggregation and oxidative damage, showing promise in preclinical studies [2]. Polypharmacology enables simultaneous modulation of multiple disease mechanisms and can also reduce medication burden and drug–drug interactions in elderly patients with multiple comorbidities [2]. As a result, polypharmacology is increasingly viewed as essential for developing disease-modifying therapies for AD and related disorders, where single-target drugs have mostly offered only symptomatic relief [2].

#### 1.3.3. Metabolic and Endocrine Disorders

Metabolic syndrome and related conditions (such as type 2 diabetes, obesity, and dyslipidemia) involve multiple interconnected abnormalities, often requiring patients to take several medications and resulting in extensive polypharmacy [18]. This complexity makes them ideal for multi-target therapeutics. Drugs that can simultaneously address glycemic control, weight loss, and cardiovascular risk are especially valuable. For instance, tirzepatide—a dual GLP-1/GIP receptor agonist—has shown superior glucose-lowering and weight reduction compared to single-target drugs [19]. Dual PPAR agonists have also been investigated to manage both lipid and glucose issues, though safety concerns emphasize the need for careful target selection. Overall, multi-target drugs can more effectively treat metabolic syndrome by addressing several aspects of the disorder at once, improving adherence, and reducing side effects compared to multiple single-target therapies [18,20].

#### 1.3.4. Infectious Diseases

Antimicrobial resistance highlights the limitations of single-target therapies, as pathogens quickly develop resistance mutations under selective pressure. Polypharmacology addresses this by enabling the design of antibiotic hybrids—single molecules that attack multiple bacterial targets, such as combining a quinolone with a membrane disruptor, thus achieving synergistic effects and reducing resistance risk since bacteria would need simultaneous mutations in different pathways to survive. Similarly, multi-target antivirals can inhibit both viral and host factors needed for replication. While combination therapies are standard (e.g., HAART for HIV or multidrug TB regimens), single multi-target agents can simplify treatment and ensure coordinated delivery. Multi-action antimicrobials may also disrupt pathogenic processes like biofilm formation or virulence, although optimizing their pharmacodynamic and pharmacokinetic profiles remains challenging. Overall, polypharmacology is emerging as an essential strategy to combat complex infections in the post-antibiotic era, where single-target drugs increasingly fail [21].

## 2. Emerging Trends and Outlook for Polypharmacology in Drug Discovery

Polypharmacology has evolved from a controversial idea into a mainstream principle driving modern drug discovery. Over the past decade, there has been a surge in research on multi-target compounds, accompanied by new methodologies to design and optimize them [10,15]. Medicinal chemists are devising innovative strategies—such as molecular hybridization, fragment linking, and structure-based polypharmacology—to intentionally craft molecules with dual or triple activities while retaining drug-like properties [10,22]. These efforts have been bolstered by advances in computational biology and systems pharmacology: network modeling and in silico off-target prediction can now guide the selection of target combinations and chemical motifs likely to yield synergistic effects. In parallel, high-throughput screening campaigns are increasingly considering multi-target readouts or phenotypic endpoints (which inherently capture multi-target effects) to identify candidate polypharmacological agents [4].

Notably, the pharmaceutical industry has started to deliver tangible outcomes from the polypharmacology paradigm. A number of multi-target drugs have reached the market or late-stage clinical trials in recent years. A recent review highlighted that among new drugs approved in 2022, many were deliberately multi-target in their mechanism—including seven oncology drugs, an antidepressant, and others—reflecting a strategic shift in drug design philosophy [9]. These include multi-kinase inhibitors for cancer, multi-receptor agonists for metabolic disease, and even CNS drugs with polypharmacological profiles optimized for complex psychiatric conditions [9]. The clinical success of agents like dimethyl fumarate (an immunomodulator with multiple molecular effects in multiple sclerosis) and clozapine (an antipsychotic hitting dopamine, serotonin, and other receptors) further validates that intelligently balancing polypharmacology can translate to superior therapeutic outcomes [1,9].

Crucially, the scientific community now recognizes that polypharmacology is not a haphazard side-effect of “dirty drugs,” but rather a feature that can be rationally harnessed. The emerging field of network pharmacology provides a conceptual framework for this: diseases are viewed as perturbations in networks, and the most effective interventions may involve modulating multiple network nodes [3,5]. This perspective aligns with systems biology and precision medicine, which acknowledge the heterogeneity and complexity of disease mechanisms. As we move forward, polypharmacology is expected to play an even greater role in addressing unmet medical needs. In areas like neurodegeneration, cancer, and antibiotic resistance, multi-target approaches are likely to yield the breakthroughs that single-target drugs have struggled to achieve.

The shift toward polypharmacology in small-molecule drug discovery represents a paradigm change driven by the realities of disease complexity and clinical experience. Polypharmacology provides a strategy to tackle the robustness of biological systems—by striking multiple targets, we can potentially circumvent compensatory mechanisms and achieve more profound, durable therapeutic effects. The introduction of artificial intelligence (AI) and machine learning into this arena (the focus of subsequent sections of this review) further accelerates the identification and design of polypharmacological agents, opening new horizons for multi-target drug discovery. The following sections will build upon this introductory foundation, exploring how AI-driven methods are revolutionizing the way we discover and optimize multi-target small molecules for polypharmacology.

## 3. Computational Approaches for Polypharmacology in Small-Molecule Drug Design

**Figure 2:**
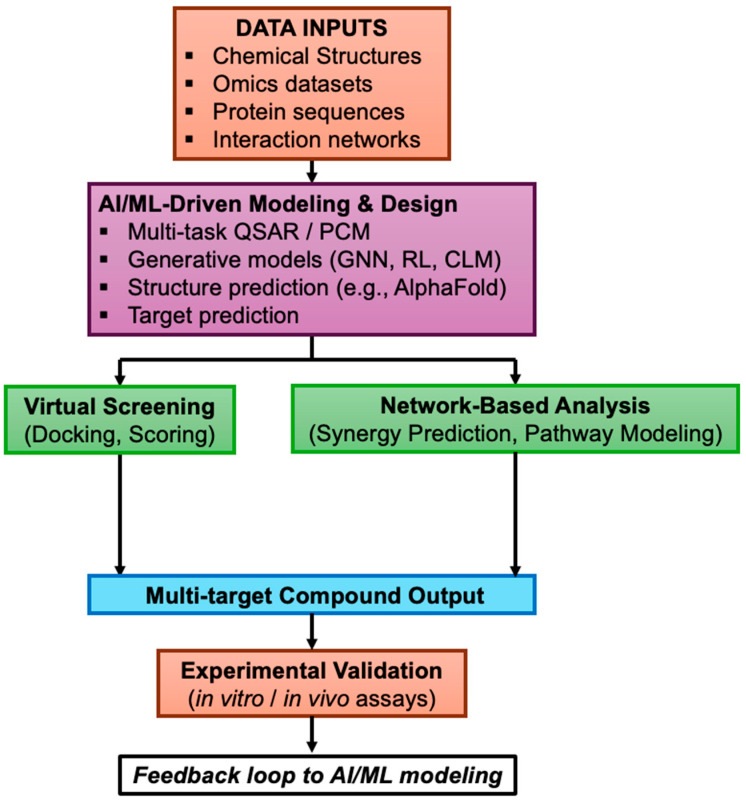
AI-driven polypharmacology workflow. This schematic illustrates a modular, iterative workflow for discovering multi-target small molecules using artificial intelligence. Data inputs (chemical, omics, protein, and network data) feed into a unified AI/ML-driven modeling hub that includes multi-target QSAR, structure prediction (e.g., AlphaFold), and generative models (deep learning, reinforcement learning, graph neural networks). Outputs are screened and analyzed via virtual docking and network-based prioritization. Promising candidates proceed to experimental validation, with results feeding back to refine AI models. This modular approach reflects the non-linear and flexible nature of AI-enabled polypharmacology.

Polypharmacology is now seen as crucial for treating complex diseases where single-target drugs often fail due to biological redundancies and resistance. Many effective drugs work by acting on multiple proteins, leading to a move away from the traditional one-target–one-drug approach. Computational polypharmacology has arisen to address the challenge of designing and predicting multi-target ligands using in silico methods to explore large chemical and target spaces (Figure 2) [20]. Below, we review key computational approaches—ligand-based modeling (multi-task QSAR and proteochemometrics), structure-based methods (molecular docking), and network-based strategies—that are being applied to identify and design multi-target small molecules. We also discuss the strengths and limitations of these approaches in a broad drug discovery context, independent of any specific disease area.

### 3.1. Ligand-Based Multi-Target Modeling: Multi-Task QSAR and Proteochemometrics

Ligand-based approaches aim to learn patterns from known compound activities to predict outcomes for new molecules, and have been extended to multi-target quantitative structure–activity relationship (QSAR) modeling. Traditional QSAR relates chemical descriptors of compounds to activity on a single biological target, but multi-task (or multi-target) QSAR methods build models that simultaneously predict activities across multiple targets. By training on cross-target data, such models can capture correlations between targets and identify chemical features contributing to polypharmacology. For example, a recent study reported a multi-target QSAR model using a multi-layer perceptron neural network that achieved > 80% accuracy in classifying inhibitors for five different parasite proteins [23]. The model’s interpretations enabled design of new molecules with broad antiparasitic activity, which were validated as multi-target inhibitors by both in silico prediction and docking simulations [23]. This illustrates how multi-task machine learning can generate de novo multi-target ligand candidates in silico.

A powerful extension of multi-target QSAR is proteochemometrics (PCM), a chemogenomic modeling approach that incorporates descriptors of both ligands and protein targets [24]. In proteochemometric modeling, one builds a single supervised learning model on a combined feature space of chemical structure properties and protein sequence/structure properties [24]. By explicitly encoding target information, PCM can generalize across protein families and predict the bioactivities of compounds against multiple related targets in one framework [24]. This approach has been used to model ligand binding profiles for entire target families (e.g., GPCRs or kinases), improving predictive power in multi-target settings [24]. Modern PCM models often employ machine learning algorithms like random forests or support vector machines, and recent work is exploring deep learning for further gains (though deep learning will be addressed in a later section). By capturing ligand–protein interactions and even ligand–protein cross-terms, PCM can predict not only the potency but also selectivity or promiscuity of a compound across a panel of targets [24]. In practice, ligand-based multi-target models (whether multi-task QSAR or PCM) are valuable for virtual screening: they can rapidly screen libraries to find molecules with desired multi-target activity profiles or flag existing drugs’ off-target activities. Early examples include similarity-based methods (e.g., the Similarity Ensemble Approach) that compare chemical similarity to sets of known ligands for various targets to predict new target hits; such ligand-centric target prediction strategies laid the foundation for computational polypharmacology [25]. Today, numerous web tools implement in silico target fishing, which aims to identify all potential targets for a given small molecule using ligand-based methods, often augmented with machine learning [25]. These approaches have become key enablers for polypharmacology and drug repurposing studies, allowing researchers to hypothesize new target interactions for molecules efficiently in silico [25]. Table 1 summarizes the principles, strengths, limitations, and application scenarios of the main computational drug screening modes.

### 3.2. Strengths and Limitations of Current Computational Techniques

Contemporary computational approaches have significantly accelerated polypharmacology research, offering the ability to explore large chemical libraries and expansive target spaces in silico. A clear strength is efficiency: methods like target fishing can quickly screen a compound against thousands of potential targets, a task infeasible to do experimentally in a short time [25]. Ligand-based multi-target models can leverage the growing volume of public bioactivity data to uncover patterns of promiscuity, even predicting off-target effects early in the drug discovery process. Structure-based docking provides structural insights that are invaluable for understanding multi-target binding and guiding medicinal chemistry modifications. Network pharmacology, on the other hand, adds a holistic view, helping to choose the right targets in the first place and potentially uncovering synergistic multi-target mechanisms that purely ligand-centric methods might miss. Together, these tools enable a more informed design of multi-target agents, and they have already shown successes such as identifying multitarget leads in infectious disease and repurposing known drugs for new indications (Table 2) [23].

Despite these strengths, there are notable limitations and challenges to address. A fundamental issue is data quality and availability: robust multi-target models demand large, high-quality datasets of compounds tested across multiple targets, yet such comprehensive data are often sparse or biased towards well-studied proteins [20]. This sparsity can limit the reliability of QSAR/PCM predictions, especially for novel targets or chemotypes outside the training data. Moreover, multi-target QSAR models can struggle to extrapolate beyond their domain—a model may accurately interpolate within known chemistry/biology space but fail when either a new scaffold or a new target is introduced. Proteochemometric models mitigate this by including target descriptors, but they require meaningful representations of protein features and, ideally, some sequence or structural similarity among targets to generalize well. Molecular docking, while broadly applicable, faces the perennial challenge of scoring function accuracy; predicting binding affinities (and ranking polypharmacology candidates) is error-prone, and false positives are common. Docking results can be particularly unreliable for targets with induced-fit binding or for ligands that undergo significant conformational changes—important considerations when one ligand may bind multiple proteins with different pocket characteristics. Additionally, docking relies on having 3D structures of all targets (or good homology models), which may not be available or may be static snapshots that do not account for dynamic plasticity.

**Figure 3:**
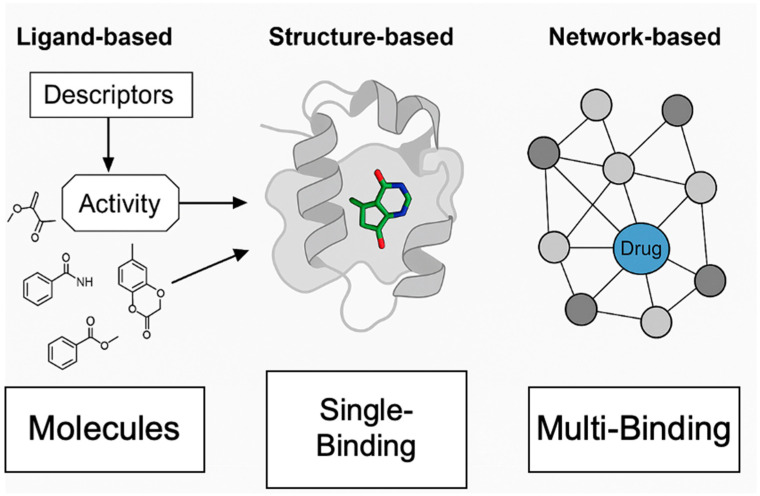
Schematic comparison of ligand-based, structure-based, and network-based drug screening. The figure presents a three-panel schematic highlighting key differences among major computational drug screening approaches. **Left** panel: Ligand-based screening is depicted by mapping chemical structures (molecules) to biological activity using molecular descriptors, without requiring protein structural information. **Center** panel: Structure-based screening illustrates a ligand docking into a protein binding site, emphasizing direct modeling of ligand–target interactions. **Right** panel: Network-based screening shows a disease network diagram, with a drug targeting multiple protein nodes, illustrating the systems-level approach for identifying multi-target interventions.

Network-based approaches come with their own caveats. Biological networks are incredibly complex and often incompletely mapped; protein–protein interaction networks or gene networks used for analysis might miss relevant interactions or include false ones. Thus, a network algorithm might prioritize a target combination based on an incomplete picture. Furthermore, network pharmacology predictions imply that modulating certain nodes will produce a therapeutic effect, but they do not ensure that a single chemical entity can drug all those nodes effectively. Bridging the gap between network predictions and actual multi-target compounds still relies on the other techniques. In general, polypharmacology design requires balancing multiple structure–activity relationships, which is inherently challenging [3]. Achieving sufficient potency on all targets while avoiding anti-targets (off-targets that cause toxicity) can lead to conflicting design requirements. Current computational methods provide guidance, but the “objective function” in multi-target optimization is complex and sometimes poorly defined (Figure 3).

Finally, a cross-cutting limitation is the lack of dedicated experimental assays and validation pipelines for multi-target effects [20]. It is often non-trivial to confirm in vitro that a single compound hits all intended targets and yields the predicted network-level outcome. This means computational hits must undergo extensive experimental validation, and negative results feed back to improve the models. Despite these challenges, progress is being made. There is a clear trend of integrating methods (e.g., using network analysis to pick target combos, then applying AI-driven molecular design to find compounds, followed by docking and molecular dynamics to refine binding modes), which together mitigate individual limitations. As noted in recent reviews, the polypharmacology field is evolving rapidly, propelled by community efforts in data sharing, multi-omics integration, and AI innovations, all of which promise to make multi-target drug discovery more tractable [20]. Nonetheless, practitioners must be aware of each technique’s limitations: predictions should be considered hypothesis-generating rather than definitive, and the strength of computational approaches lies in focusing experimental resources on the most promising multi-target candidates rather than replacing experiments altogether.

## 4. Generative and AI Methods

Recent advances in AI offer new avenues to tackle the challenge of crafting a single compound with potent activity at several distinct proteins. Deep learning and generative models can learn chemical features associated with bioactivity and explore vast chemical spaces in a directed manner, enabling de novo design of multi-target ligands. Early successes of deep generative models in drug discovery—for example, designing novel kinase inhibitors in days rather than years [27]—have paved the way for extending these methods to polypharmacology. In this section, we review how AI-driven techniques, especially deep generative models, graph neural networks, and reinforcement learning, are being applied to design and optimize small molecules with desired multi-target profiles. We also discuss deep learning approaches for predicting off-target effects, and highlight emerging tools (e.g., POLYGON) and case studies where AI-designed polypharmacology compounds have been experimentally validated.

### 4.1. Generative Deep Learning for Polypharmacology

Generative deep learning models have demonstrated the ability to design novel molecules with specified biological activities [26]. Most conventional models were trained to generate compounds for a single target or objective. Recently, researchers have adapted generative frameworks to explicitly handle multiple targets. One approach is to use chemical language models (CLMs), which treat molecules as text sequences (SMILES strings). CLMs can be pretrained on large chemical libraries and then fine-tuned on a small set of known ligands for each target of interest [26]. Isigkeit et al. (2024) applied a CLM to multi-target design by fine-tuning on ligand sets for six target pairs, thereby biasing the model toward regions of chemical space that overlap known binders of both targets [26]. This approach successfully generated candidate dual ligands: in a prospective test, twelve AI-designed molecules were synthesized for various target pairs, and all showed activity on at least one intended protein (seven compounds were confirmed as dual-acting, with potencies in the low nanomolar range) [26]. These results underscore that generative models can capture combined pharmacophore requirements and produce innovative chemotypes for polypharmacology [26].

Another strategy is to incorporate multiple biological objectives into reinforcement learning (RL)-based generators. In RL frameworks for molecule design, an AI agent modifies a molecule stepwise (through SMILES characters or graph edits) and receives a reward based on predicted properties. By shaping the reward to include multiple targets, the agent can be directed toward polypharmacology solutions. For example, Liu et al. (2021) developed DrugEx v2, an RNN-based RL system extended for multi-objective optimization [28]. In their study, the agent was rewarded for generating molecules that activate two desired receptors (A_1 and A_2A adenosine receptors) while avoiding an anti-target (the hERG ion channel associated with cardiac toxicity) [28]. A Pareto-based reward scheme ensured a balanced optimization across objectives, yielding diverse compounds with predicted selective polypharmacology profiles [28]. This demonstrates how RL can navigate trade-offs between efficacy and safety targets in a single de novo design loop.

Likewise, graph-based generative models have been harnessed for polypharmacology. Graph neural networks (GNNs), which represent molecules as node–edge graphs, are well-suited to capture structural features relevant to binding multiple proteins. Mukaidaisi et al. (2022) combined a graph-fragment generative model with an evolutionary algorithm to carry out large-scale multi-objective molecular design [29]. Their method optimized compounds against protein–ligand affinity predictions for multiple targets alongside other drug-like properties [29]. By integrating protein binding scores directly into the generation process, such approaches ensure that the proposed molecules are not only generally “drug-like” but specifically tailored to engage multiple targets. Overall, generative AI methods—whether based on sequence models, GNNs, or hybrid schemes—have opened a path to systematically explore polypharmacology space that was previously intractable by intuition or trial-and-error.

Notably, several emerging frameworks are dedicated to multi-target generation. POLYGEN (Polypharmacy Generative Network) and related architectures leverage variational autoencoders (VAEs) or transformers coupled with custom reward functions to embed chemical space for multi-target objectives [30]. POLYGON (Polypharmacology Generative Optimization Network) is a prime example: developed by Munson et al. (2024), POLYGON uses a VAE to embed molecules into a latent space and applies generative RL to sample new structures, rewarding candidates that are predicted to inhibit each of two protein targets simultaneously [27]. This model was trained on large binding datasets and could correctly recognize known dual-target interactions with ~82% accuracy. POLYGON then generated novel compounds for ten different target pairs with documented biological synergy. Impressively, when Munson et al. synthesized 32 top-ranked POLYGON designs for one pair (MEK1 and mTOR kinases), most compounds showed >50% inhibition of both targets in enzymatic assays and reduced cancer cell viability at low micromolar doses [27]. Such dual inhibitors, identified in silico and confirmed in vitro, validate the power of generative AI to deliver functional polypharmacology molecules. Similarly, MTMol-GPT proposed by Ai et al. (2024) is a transformer-based generative model that employs an imitation learning paradigm to concurrently optimize for multiple targets [31]. MTMol-GPT was demonstrated on cases like EGFR and Src kinases and dopamine and serotonin receptors, producing novel candidates with predicted dual activity; follow-up docking studies suggested these AI-generated molecules could indeed bind both intended targets [31]. These cutting-edge tools underscore a trend in which de novo drug design is evolving from single-target optimization toward target polyphony, guided by AI.

### 4.2. Graph Neural Networks and Multi-Objective Reinforcement Learning

Graph neural networks (GNNs) have become a cornerstone of AI-driven chemistry, owing to their ability to learn rich representations of molecular structures. In polypharmacology contexts, GNNs are employed in two main ways: (1) as encoders within predictive models that evaluate a molecule’s activities on various targets, and (2) as generative models or policy networks that construct new molecules. The strength of GNNs lies in capturing substructural features (e.g., functional groups, ring systems) that contribute to binding at different protein sites. For instance, message-passing neural networks can learn that a molecule containing a certain scaffold may simultaneously fit kinase ATP-binding pockets and a GPCR ligand site, if such patterns exist in the training data. This capability is valuable for multi-target design, where subtle structural compromises are needed to engage distinct proteins.

When coupled with reinforcement learning, GNNs enable sophisticated multi-objective optimization. In an RL setting for drug design, a GNN-based agent can propose molecular modifications and use a learned reward function to decide beneficial moves. Multi-objective RL extends this by formulating the reward as a composite of several goals—e.g., activity against Target A, activity against Target B, and ADMET properties. Early demonstrations of deep RL in drug discovery, like the work of Popova et al. (2018) on de novo drug design, showed that an AI agent could be trained to optimize simple objectives (such as binding to one target or improving drug-likeness) [32]. Now researchers are generalizing these agents to tackle simultaneous objectives. In practice, a multi-objective RL agent might receive a positive reward if a generated molecule is predicted (by a fast proxy model) to be active on all desired targets and also meets thresholds for pharmacokinetic properties. If the agent uses a graph-based state representation, it can intelligently navigate chemical modifications that improve, say, target selectivity without introducing toxicophores.

One challenge in multi-objective RL is balancing competing criteria. Techniques from Pareto optimization and evolutionary algorithms have been integrated to address this [28]. For example, the DrugEx v2 system mentioned earlier ranks each batch of generated molecules by Pareto dominance (non-dominated sorting) to identify those compounds that present the best trade-off between objectives [28]. The agent’s reward is then adjusted based on Pareto rank rather than a single weighted sum, which encourages diversity in solutions that excel in different aspects. Such schemes prevent the collapse of the generator towards only one extreme of the multi-objective space. In the DrugEx v2 case, after training, the RL agent was able to output molecules that showed predicted affinity for both A_1 and A_2A receptors while minimizing predicted hERG activity—essentially learning to avoid a key off-target without sacrificing on-target potency [28].

Beyond reinforcement learning, conditional generative models using GNNs also facilitate multi-target optimization. Conditional graph generative models can accept target-specific context as input (for instance, a protein embedding or a desired polypharmacology profile) and then generate molecules conditioned on that context. This approach is being explored to design molecules on demand for arbitrary target combinations. For example, a recent study introduced a multitask framework that intertwines drug–target affinity prediction with molecule generation [33]. In this model, a shared GNN learns the underlying chemical features important for binding, and the generative component produces new molecules while being “aware” of the target by construction [33]. Such target-aware generation ensures that the output molecules are not generic drug-like compounds but are structurally tuned to the chosen protein(s). This is particularly relevant for polypharmacology, where a generative model might be tasked with designing a molecule for a specific combination of targets (e.g., a kinase and a nuclear receptor)—a scenario where conditioning on both protein contexts is crucial. Although this is an emerging area, the synergy of GNNs for molecular representation and multi-objective RL/conditional generation is a powerful paradigm for refining leads that hit multiple targets while satisfying medicinal chemistry constraints.

### 4.3. Emerging Tools and Case Studies

The convergence of generative models and multi-target predictions has led to a new generation of computational tools for polypharmacology. One prominent example, mentioned earlier, is POLYGON [27]. POLYGON’s framework (generative RL with dual-target rewards) not only produced virtual hits but was taken through the full experimental cycle [27]. Munson et al. reported synthesizing dozens of POLYGON-suggested molecules for various target pairs—including *EGFR + PI3K*, *BCL2 + PI3K*, and *MEK1 + mTOR*—based on hypotheses of network co-dependencies in cancer. These AI-designed compounds were then tested in cell-based assays, where several showed the intended polypharmacological activity and downstream biological effects. Such studies represent a milestone: they demonstrate that AI can generate hypothesis molecules for polypharmacology that are novel, synthesizable, and biologically viable. In the case of the MEK1/mTOR dual inhibitors, for example, the best POLYGON compound achieved sub-micromolar potency on both kinases and inhibited proliferation in a cancer cell line, validating the AI’s design [27].

Another case study comes from Merk and colleagues (2024), who employed a fine-tuned CLM to design multi-target ligands as described above [10]. They focused on target pairs relevant to metabolic syndrome (e.g., a GPCR plus a nuclear receptor involved in glucose regulation). After generating candidate molecules in silico, they synthesized a set of top candidates. Experimental testing confirmed that more than half of these compounds were active on both intended targets, in some cases with low-nanomolar potency [26]. This outcome is remarkable given that the model had seen only a few dozen dual-target template molecules during fine-tuning. It suggests that the model extrapolated novel analogs that successfully bridged the chemistry of two distinct ligand families. The authors highlighted one dual ligand that activated PPARδ while antagonizing the angiotensin II receptor AT1—a combination aimed at synergistically addressing cardiovascular and metabolic aspects of disease. This illustrates how AI can generate non-intuitive polypharmacological solutions that human designers might overlook.

Emerging platforms also integrate structure-based insights. For example, some recent tools incorporate AI-guided docking evaluations within the generative loop: after proposing a molecule, the platform uses a fast neural network proxy for docking or binding affinity to multiple targets, feeding that score back into the design model [34]. In principle, this marries the strengths of structure-based drug design (accurate consideration of binding geometry) with the speed of deep learning. Although classical docking is beyond our scope here, its AI-accelerated variants are increasingly used to validate multi-target binding poses for generated compounds. In the POLYGON study, retrospective docking was used to show that the AI designs could fit into both target binding sites in orientations similar to known single-target drugs [27]. Meanwhile, diffusion models and transformer-based inverse design are emerging as alternatives to RL for generating polypharmacology candidates, sometimes yielding better novelty or synthetic accessibility. Regardless of the specific algorithm, the trend is clear: AI systems are growing more adept at concurrently handling the multiple objectives (biological and chemical) inherent in polypharmacology.

In summary, the literature of the past decade (2015 onward) reflects a rapid expansion of AI-driven methods for multi-target small-molecule design. From deep neural networks that predict broad target profiles, to generative models that de novo create dual or multi-target ligands, these tools are transforming polypharmacology from a serendipitous art into a rational design strategy. Early validation studies—including AI-designed dual inhibitors progressing to experimental testing—lend credence to the approach. Moving forward, challenges remain in further improving the accuracy of target prediction (especially for less-characterized proteins), incorporating pharmacokinetic and toxicity endpoints into multi-objective design, and ensuring synthetic feasibility. Nonetheless, AI-driven polypharmacology has firmly taken root as a promising frontier in drug discovery, poised to deliver innovative therapies for complex diseases that elude single-target treatments.

To provide a practical overview for readers, Table 3 summarizes the key features, methodological foundations, and application domains of the main AI-driven polypharmacology tools discussed in this review, as well as several additional platforms relevant to multi-target drug discovery.

## 5. Systems Biology and Network Pharmacology in Polypharmacology

Modern drug discovery increasingly recognizes that complex diseases cannot be effectively treated with the traditional “one drug–one target” paradigm. Highly selective single-target drugs often show limited efficacy in multifactorial diseases, as these conditions involve diverse pathways and compensatory mechanisms [35]. Consequently, there has been a paradigm shift toward network pharmacology, a systems biology approach that views drugs in the context of the entire network of molecular interactions. This holistic strategy acknowledges that many effective drugs hit multiple targets and that multi-target or combination therapies can yield synergistic benefits [35]. By considering the interplay of signaling pathways and feedback loops, network pharmacology informs polypharmacology (the design of drugs acting on multiple targets) to tackle network-level disease vulnerabilities rather than single molecular nodes. Early proponents of this approach noted that network-based drug development could better address complex diseases, reducing unanticipated off-target effects that arise from a narrow focus [35]. Indeed, multi-target agents have the potential to improve the balance between efficacy and safety, as modulating multiple disease-relevant targets can produce a more robust therapeutic effect at lower individual target potencies [36]. Such polypharmacology strategies, when guided by systems-level understanding, may also preempt drug resistance by blocking alternate pathological pathways. However, realizing this potential requires integrating vast biological knowledge—from protein–protein interaction networks to signaling cascades—into the drug design process. Notably, systematic approaches for selecting optimal target combinations remain challenging, underscoring the need for more comprehensive network models and data integration [36]. Overall, systems biology and network pharmacology provide a crucial foundation for polypharmacology by identifying network “hub” nodes and pathway crosstalk that inform which combinations of targets might yield synergistic therapeutic effects across diverse disease contexts [35].

### 5.1. Omics-Driven Target Identification for Multi-Target Drug Design

The advent of high-throughput omics technologies (genomics, transcriptomics, proteomics, metabolomics, etc.) has vastly expanded our view of disease networks, enabling data-driven identification of multiple potential targets. Single-omics studies, while useful, often capture only one layer of a complex biological system and cannot fully explain how drug perturbations lead to phenotypic outcomes [37]. In contrast, integrated multi-omics analyses provide a systems-level picture of disease processes, revealing how genetic variants, gene expression changes, protein signaling networks, and metabolic alterations converge in pathogenesis. Recent efforts have shifted toward integrative multi-omics techniques for target discovery, which can highlight key nodes that might be missed by any single data type [37]. For example, combining cancer genomics with proteomic and phospho-proteomic data can pinpoint signaling proteins that are not only genetically altered but also abnormally activated, suggesting they could be dual hits for therapy. By mapping disease “modules”—sets of interacting or co-regulated molecules—researchers can identify combinations of targets that collectively disrupt a pathogenic network. An omics-driven network view often reveals that tackling a disease module may require inhibiting multiple proteins within a pathway or in parallel pathways. Importantly, multi-omics profiling can distinguish drivers from passengers in disease networks, thereby prioritizing targets most critical to phenotype. It also enables the discovery of polypharmacology profiles of existing drugs; for instance, transcriptomic and proteomic profiling of cells exposed to a drug can uncover off-target effects or pathway adaptations, informing the design of new compounds that either exploit those effects or avoid them. Integrated omics has become instrumental in proposing multi-target strategies across disease areas—from cancer to neurological disorders—by uncovering pathway co-dependencies. As one illustration, a comprehensive multi-omics study of CDK4/6 inhibitor response in cancer cells revealed networks of downstream and off-target effects, suggesting additional nodes (beyond CDK4/6) that a polypharmacological drug might modulate to improve efficacy [20]. In summary, omics-driven target identification aligns with systems biology by providing the rich datasets needed to choose target combinations rationally. This approach increases the likelihood that multi-target drugs hit the most pivotal points in a disease network, rather than just the most obvious single gene or protein. Ongoing advancements in proteogenomics, single-cell omics, and network-based multi-omics integration are further refining our ability to design multi-target interventions guided by molecular data [37].

### 5.2. Functional Genomic Screens and Network Pharmacology

While omics data can suggest correlations and network associations, functional genomic screens (such as RNA interference and CRISPR-Cas9 knockout/knockdown screens) directly interrogate the causal roles of genes, thereby complementing network pharmacology by revealing which nodes are truly actionable vulnerabilities. CRISPR screening in particular has revolutionized target discovery by enabling systematic loss-of-function studies across the genome. In the context of polypharmacology, researchers are leveraging CRISPR not only to find single gene targets, but also to uncover synergistic interactions between genes. For example, genome-wide CRISPR dropout screens can highlight multiple genes whose concurrent disruption yields synthetic lethality in cancer cells, indicating that a drug hitting both would have enhanced efficacy. Pairwise or combinatorial CRISPR screens go a step further by simultaneously perturbing gene pairs: these approaches have identified gene pairs that are synthetically lethal or synergistic, pointing to combinations of targets for potential dual-target therapies. A recent combinatorial CRISPR study in triple-negative breast cancer (TNBC) cells demonstrated this approach: by knocking out pairs of tyrosine kinases, investigators discovered a synergistic interaction between FYN (a Src-family kinase) and KDM4 (a histone demethylase)—dual inhibition of which greatly enhanced the response to multiple kinase inhibitors [38]. Mechanistic analysis showed that cancer cells upregulated KDM4 and FYN in response to kinase inhibitor treatment as a resistance mechanism; thus, jointly targeting these compensatory nodes could overcome drug resistance [38]. More broadly, CRISPR-based screens have been used to map genetic interactions and identify “co-essential” gene sets in cancer and other diseases. These functional datasets, when integrated with network models, help validate which network connections are critical for cell survival or disease progression. In network pharmacology terms, CRISPR screens can empirically confirm the importance of network hubs or validate predicted multi-target combinations. They also enable the discovery of anti-targets—genes whose loss exacerbates toxicity or other undesirable effects—which polypharmacology efforts should avoid. For instance, integrating cell-based drug sensitivity screens with CRISPR loss-of-function data has helped pinpoint which genes, when inhibited together, produce a therapeutic effect versus those whose combined inhibition leads to cell death or toxicity [39]. Such knowledge guides multi-target drug design toward synergistic co-targets and away from hazardous target combinations. In summary, functional genomic screens provide a powerful experimental pillar for network pharmacology by identifying and validating target networks. As large CRISPR screening data accumulates, AI models (discussed later) are beginning to mine these data to predict effective multi-target interventions. In the meantime, CRISPR and related techniques are increasingly used to prioritize target sets from hundreds of initial candidates, ensuring that polypharmacological strategies focus on functionally relevant nodes in the system.

### 5.3. Pathway Modeling and Simulation for Multi-Target Design

Beyond experimental data, computational pathway simulations serve as important tools in designing multi-target therapeutics. Systems biology offers a variety of modeling formalisms—from logic-based networks to ordinary differential equation (ODE) models—that can simulate the dynamic behavior of biological pathways under different perturbations. By constructing in silico models of disease-relevant networks, researchers can virtually “test” the effect of hitting multiple targets simultaneously, thereby guiding hypothesis generation for polypharmacology. For example, cell signaling networks involved in cancer (such as MAPK or PI3K/AKT pathways) have been modeled to understand how inhibiting two nodes might produce synergistic shutdown of an oncogenic signal. These simulations can reveal non-linear effects: in some cases, dual inhibition of two partially redundant pathways produces a dramatic effect that neither alone could achieve; in other cases, targeting two nodes might be antagonistic if one inhibition activates a compensatory loop that the second target lies on. Network pharmacology modeling frameworks like TIMMA (Target Inhibition network modeling) explicitly attempt to predict optimal target combinations by integrating drug sensitivity data with network topology [40]. In one study, a network model was used to predict synergistic drug pairs in TNBC, which were then tested experimentally. The model successfully anticipated essential target pairs, later confirmed by combinatorial gene knockdowns [40]. Furthermore, after identifying a particular synergistic target pair (Aurora B kinase and ZAK kinase) via modeling, researchers employed dynamic pathway simulation to explore why inhibiting those two kinases yielded synergy in cancer cells [40]. The simulation of the cancer signaling network showed that dual Aurora B/ZAK inhibition concurrently disrupted mitotic checkpoints and stress response pathways, explaining the strong anti-proliferative effect. This mechanistic insight, obtained in silico, suggested that a single drug hitting both kinases or a combo therapy could be especially effective in certain patient subsets [40]. Similarly, rule-based multi-scale simulations have been applied in other contexts (e.g., metabolic diseases) to evaluate multi-target interventions across different biological levels, from molecular interactions up to organ systems [41]. Such simulations underscore that treating complex diseases often requires multi-target modulation at multiple scales. While constructing accurate multi-scale models is challenging due to data gaps, even pathway-focused models can highlight crucial feedback loops and cross-talk. For instance, computational perturbation biology methods have inferred how crosstalk between parallel pathways can cause drug resistance, thereby suggesting that both pathways should be co-inhibited [40]. In summary, pathway and network simulations act as a “dry lab” for polypharmacology, allowing researchers to prioritize which multi-target hypotheses are worth pursuing in wet-lab experiments. By integrating known biochemical kinetics and network architectures, simulations can guide the design of multi-target compounds that yield desired network-level outcomes (e.g., inducing apoptosis or halting cell cycle) while minimizing unintended pathway activations. The predictive power of these models will continue to grow as they incorporate more high-quality omics and kinetic data, and as AI techniques are applied to calibrate and refine them.

### 5.4. Risks and Limitations of Current AI Approaches

While artificial intelligence techniques (e.g., machine learning and deep learning) have yielded impressive advances in polypharmacology modeling [20], they come with notable limitations and risks. A foremost concern is model bias and generalizability. AI models are only as good as the data they learn from; if multi-target training data are biased toward well-studied protein families or chemotypes, the models will preferentially excel in those areas and might falter on less-represented target classes. For example, models trained on abundant kinase inhibitor data may poorly predict multi-target ligands for GPCRs or novel enzymes. Similarly, many AI polypharmacology models risk overfitting to limited datasets—they may appear accurate on known drug–target profiles but fail to extrapolate to truly novel target combinations or chemistries. This undermines their utility in discovering unexpected polypharmacological effects.

Another limitation is the reproducibility and transparency of AI predictions. Deep learning models often operate as “black boxes” with complex architectures, making it difficult for researchers to interpret why a multi-target prediction was made. This opaqueness can erode trust, especially in safety-critical decisions like flagging off-target liabilities. In drug discovery, there is growing concern about the reproducibility crisis and the need to carefully vet computational findings [42]. Many AI models in drug discovery yield promising results in academic settings, but translating these into robust, validated tools for industry use remains problematic. Without sufficient “ground truth” data and careful human oversight, AI systems may output false positives (e.g., predicting a spurious target interaction) or false negatives (missing a crucial off-target) [42]. Over-reliance on such models carries the risk of misdirecting experimental efforts—for instance, pursuing a predicted multi-target mechanism that in reality is ineffective or unsafe. Consequently, experts emphasize that AI-driven predictions must be complemented by human expert review and experimental validation to mitigate these risks [42]. Additionally, current AI approaches often neglect the dynamic and context-dependent nature of polypharmacology. In vivo, the efficacy and toxicity resulting from multi-target modulation depend on tissue context, feedback loops, and network adaptations that static in silico models may not capture. This raises the risk that a compound predicted to have an ideal multi-target profile in silico might behave unexpectedly in a complex biological system. Therefore, the deployment of AI in polypharmacology must contend with these limitations, ensuring models are robust, interpretable, and used with appropriate caution in the drug discovery pipeline.

## 6. Conclusions

Looking ahead, the convergence of these trends is expected to significantly advance AI-driven polypharmacology over the next decade. In research pipelines, we will likely see more routine discovery of multi-target drug candidates informed by AI. The improvement of multi-target datasets—via automated experiments and collaborative data-sharing—will yield more robust and generalizable AI models. These models, in turn, should become more interpretable, aided by techniques in explainable AI, so that chemists and biologists can understand the rationale behind predicted target profiles and confidently act on them. Importantly, future models may better account for systems-level effects, perhaps by integrating network simulations or cell-specific contexts, thus narrowing down the translational gap between in silico predictions and in vivo outcomes. The notion of “self-driving drug discovery” may become reality: AI-guided platforms continuously designing, testing, and learning, dramatically shortening the cycle to identify efficacious polypharmacological compounds. Such approaches could be especially impactful in drug repurposing and personalized medicine. By leveraging polypharmacology, AI could find new uses for existing drugs (identifying secondary targets that modulate other disease pathways) or tailor multi-target drug combinations suited to an individual patient’s molecular profile. Indeed, combination therapies and multi-target drugs are expected to grow in importance for complex diseases, and AI will be central in pinpointing optimal combinations while minimizing adverse interactions.

## 7. Future Directions

On the clinical translation front, the next 5–10 years could witness the first AI-designed polypharmacology drugs entering clinical trials. Early examples of compounds suggested by AI with dual or multiple targets will test the real-world efficacy of this paradigm. Successes will likely spur greater acceptance of AI in regulatory and pharmaceutical communities. We anticipate that regulatory agencies will develop clearer guidelines for AI-developed molecules and perhaps begin to incorporate AI predictive evidence (for example, predictive toxicology from off-target models) into their evaluation process. Clinically, a validated ability to intentionally hit multiple targets could translate into therapies with improved efficacy for refractory diseases (e.g., cancers that evade single-agent treatments, neurodegenerative diseases with multifactorial pathology) as well as better safety profiles achieved by dialing out undesirable off-targets early in development [43]. However, with these advances, maintaining a cross-disease perspective will be important—ensuring that AI frameworks are generalized and not limited to just a few therapeutic areas. If current trends continue, polypharmacology will evolve from a niche concept into a mainstream drug design strategy supported by AI at every step. The field is poised to move from retrospective analyses of promiscuous drugs to the prospective, rational design of multi-target therapeutics. In sum, AI-driven polypharmacology is on course to mature substantially in the coming decade, transforming both the research approach to multi-target drug discovery and, ultimately, yielding novel treatments that tackle diseases via multiple levers for the benefit of patients.

## Figures and Tables

**Table 1:** Comparison of computational drug screening modes.

Method	Principle/Approach	Key Strengths	Limitations	Typical Application Scenarios
Ligand-based	Uses known ligands’ chemical features to predict new actives (QSAR, PCM)	Fast; no target structure needed; leverages existing bioactivity data	Limited by data quality/coverage; struggles with novel targets	Virtual screening, off-target prediction, drug repurposing [7,8]
Structure-based	Uses 3D structures of protein targets to dock and score ligands	Provides structural insights; suitable for novel chemotypes	Requires accurate protein structures; docking/scoring errors	Lead optimization, binding mode analysis, novel target screening [8,9]
Network-based	Integrates biological networks to identify target combinations	Captures system-level effects; can suggest synergistic targets	Networks often incomplete; translation to chemistry is nontrivial	Target prioritization, multi-target design, systems pharmacology [7,8,9]

**Table 2:** Typical application scenarios and example tools for each screening mode.

Screening Mode	Example Methods/Tools	Example Application/Case Study	Reference(s)
Ligand-based	Multi-task QSAR, Proteochemometrics, Similarity Ensemble Approach	Classification of antiparasitic inhibitors, target fishing	[7,8,23,24,25]
Structure-based	Molecular docking, Homology modeling, Structure-based virtual screening	Multi-target kinase inhibitor design, binding mode prediction	[8,9,26]
Network-based	Network pharmacology models, Omics integration, CRISPR screening	Synergistic target identification, pathway simulation	[13,14,15,16]

**Table 3:** Summary of Representative AI Platforms and Tools for Multi-Target Drug Discovery and Design.

Tool/Platform	Main Functionality	Method/Algorithm	Application Domain(s)	Notable Features	Reference
POLYGON	Generative design of multi-target ligands	VAE + RL	Oncology, kinase inhibitors	Dual-target optimization, experimental validation	[11,27]
MTMol-GPT	Multi-target molecule generation	Transformer + Imitation Learning	Kinases, CNS	Conditional generation, novelty	[11,31]
DrugEx v2	Multi-objective molecule design	RNN + Pareto RL	GPCRs, ADMET	Pareto optimization, anti-target avoidance	[11,28]
DeepDTAGen	Affinity prediction and target-aware generation	Multitask Deep Learning	Multiple protein classes	Integrated affinity and generation	[11,33]
Chemprop *	Target activity prediction	Message Passing Neural Net	Polypharmacology, ADMET	User-friendly, open source	-
DeepChem *	General molecular ML platform	Multiple (GNNs, DL, etc.)	Broad: prediction, generation	Extensive library, tutorials	-
